# Minimally invasive unicompartmental knee replacement: Midterm clinical outcome

**DOI:** 10.1371/journal.pone.0176082

**Published:** 2017-05-04

**Authors:** Shaoqi Tian, Jiangjun Liu, Wanqing Yuan, Yuanhe Wang, Chengzhi Ha, Lun Liu, Qicai Li, Xu Yang, Kang Sun

**Affiliations:** Department of Orthopaedics, the Affiliated Hospital of Qingdao University, Qingdao, China; Northwestern University Feinberg School of Medicine, UNITED STATES

## Abstract

**Objective:**

The purpose of this study was to explore the midterm clinical outcomes of unicompartmental knee replacement (UKR) for medial knee arthropathy through a minimally invasive approach (MIA).

**Methods:**

From January 2006 to June 2010, 442 consecutive patients (485 knees) were included in the study. All patients underwent MIA-UKR with the mobile bearing Oxford phrase III prosthesis. The incision was made starting 1 cm medial to the medial pole of the patella and extending distally to the tibial tubercle. Radiographic evaluations include femorotibial angle (FTA) from coronal x-rays and rectified varus deformity angle, while clinical evaluations included Knee Society Score (KSS, clinical score and function score), the Western Ontario and McMaster Universities Arthritis Index (WOMAC) osteoarthritis index and visual analog scale (VAS) for pain. Patients followed-up at 1, 3, 6, 12 months after surgery and each year thereafter.

**Results:**

Four hundreds and two patients completed the entire follow-up, 40 patients (45 knees) were lost to follow-up. The average follow-up time was 73.0 ± 1.9 months. The mean length of the incisions was 5.0 ± 0.2 cm. The average FTA decreased from 183.6° ± 5.1° preoperatively to 174.3° ± 4.2° postoperatively, and the mean rectified varus deformity angle was 9.3° ± 1.2°. The KSS clinical score improved from 42.4 ± 2.9 to 92.9 ± 3.8, and the function score improved from 53.5 ± 3.8 to 93.5 ± 4.0. The WOMAC score improved from 47.5 ± 3.1 preoperatively to 12.3 ± 1.5 at the last evaluation. The VAS dropped from 7.8 ± 1.9 preoperatively to 1.6 ± 0.2 postoperatively. All clinical evaluations (KSS, WOMAC, VAS) were significantly different (p < 0.05) from pre and post-operative evaluations. The survival rate was 99.1% at 73 months, and the revision rate was 0.9%.

**Conclusion:**

The midterm clinical outcomes of MIA-UKR are satisfactory in a Chinese patient population, which is a good surgical option for patients with medial arthropathy of the knee. However, longer-term follow-up studies should be performed in these patients.

## Introduction

In 1973, the first study using unicompartmental knee replacement (UKR) for the treatment of knee osteoarthritis (OA) was reported by Marmor. Since then, there is still controversy regarding the role of UKR in the treatment of OA for more than four decades [1–2]. Recently, there have been improved clinical results using UKR with improved fixation techniques, newer prosthesis design, precise surgical technique and instrumentation, increased wear resistance of polyethylene (PE), and an improved understanding of knee kinematics and indications for UKR. In the past, UKR were performed with traditional incisions used during total knee arthroplasty. Minimally invasive approaches (MIA) have been more recently utilized and are differentiated by traditional approaches. While other studies have compared traditional to MIA [3–5], those studies have not report on the outcomes of MIA-UKR in Chinese patients. Thus, the purpose of this clinical study was to report the midterm clinical outcomes of MIA-UKR for Chinese patients with medial arthropathy of the knee.

## Methods and materials

### Patients and demographic data

A retrospective study was conducted from January 2006 to June 2010, where 442 consecutive patients (485 knees) with medial compartment knee arthropathy were included in the study. Patients were included if they had: (1) medial compartment OA or idiopathic necrosis of bone, (2) intact cruciate ligaments, (3) no medial or lateral subluxation, (4) well preserved lateral compartment with an intact meniscus and full thickness articular cartilage, (5) varus deformity ≤ 15°, (6) passively correctable malalignment of the limb to neutral and not beyond, (7) flexion deformity < 15°, (8) and knee flexion to at least 110° under anesthesia. Patients were excluded if they had inflammatory arthritis, such as rheumatoid arthritis, were lost to follow-up, or had severe neuromuscular abnormalities which could affect the recovery of the operated knee joint. Four hundred**s** and two patients (440 knees) completed follow-up. The mean follow-up time was 73.0 ± 1.9 months (range 60–112 months). Forty patients (45 knees, 9%) were lost to follow-up for the following reasons: refusal to continue the study (28), unable to reach the patient due to changed home address (6) and/or phone numbers (7), and death due to other disease (4).

All eligible patients underwent MIA-UKR with Oxford phase III prosthesis (Biomet, Bridgend, UK). The Affiliated Hospital of Qingdao University’s institutional review board (IRB) approved the project and the written informed consent was obtained from the patients and documented by IRB. The following x-rays were obtained: antero-posterior (AP) radiographs in 15° flexion, lateral, femoral-patellar in 30° flexion view, and long-leg standing radiographs. Varus deformity of the knee prior to surgery and the valgus degree after surgery were measured and recorded. Stress radiographs in valgus were occasionally obtained to verify the presence of a well-preserved lateral compartment. Magnetic resonance imaging (MRI) was not performed on a regular basis. The following demographic information was obtained from patients: age, gender, and body mass index (BMI).

### Surgical procedure and postoperative managements

The surgeries were carried out according to the operation manual of Oxford phase III MIA-UKR [6–7]. All surgeries were performed by the same experienced senior surgeon. Antibiotics were administered prior to and after surgery. An incision was made starting 1 cm medial to the medial pole of the patella and extending distally to the tibial tubercle. A drain was placed for no more than 24 hours. Analgesic and anticoagulation drugs were used routinely. On the 2^nd^ day after surgery, x-rays of the operated knee were taken. 6 hours after surgery, patients performed quadriceps exercises and straight leg raises. Additionally, patients began to walk with the assistance of a walker or crutches to allow for partial weight bearing. After discharge, full weight bearing began 2 weeks after surgery.

#### Follow-up and evaluations

Follow-up was performed at 1, 3, 6, 12 months after surgery and each year thereafter. X-rays were taken for evaluation, and complications and revisions were recorded. Complications included intraoperative ligament injury, fracture, residual bone cement in the joint, postoperative collapse of the tibial plateau, infection, thrombosis, and aseptic loosening. Revisions were defined as further operative intervention, including change of prosthetic components or dislocation. Clinical evaluations included the Knee Society Score (KSS)-clinical score and function score (Excellent, score 85–100, Good, score 70–84, Fair, score 60–69, Poor, score below 60), the Western Ontario and McMaster Universities Arthritis Index (WOMAC) osteoarthritis scores (score 0–96) and visual analog scale (VAS) (score 0–10). Serial radiographs were assessed for evidence of component loosening, the presence, type and extent of radiolucencies, and progression of OA in the lateral compartment. Radiographs were reviewed independently by two observers who were blinded to the clinical outcomes. Other evaluations were done by two fellowship-trained orthopedic surgeons who were also blinded to the study.

Radiolucencies were analyzed and classified as pathological or physiological based on descriptions given by Goodfellow et. al. [8–9]. A physiological radiolucency was defined as being a non-progressive radiolucency less than 2 mm (usually 1 mm) in thickness with a sclerotic margin, in contrast to a pathological radiolucency that is progressive, poorly defined and more than 2 mm thick. Subsidence was considered to be evidence of loosening. This was assessed by comparing the latest set of radiographs with immediate post-operative radiograph and examining for changes in component position.

### Statistical analysis

Statistical analyses were performed using SPSS for Windows (version 18.0; SPSS Inc., Chicago, Illinois, USA). The data were found not to be normally distributed and were therefore analyzed using Wilcoxon’s signed ranks test (WSR) for discrete data and the Mann-Whitney U test for continuous data. Survival, defined as time to revision, was assessed using the method described by Peto et. al. [10]. Interobserver reproducibility was assessed on serial radiographs by the unweighted κ statistic. The inter-observer reliability for radiological assessment was also examined. The significant level was set at p ≤ 0.05.

## Results

Of the 402 patients who completed follow-up, there were 165 male patients (177 knees) and 237 female patients (263 knees), 232 left knees and 208 right knees. As to the diagnosis, there were 427 knees with the diagnosis of medial compartment OA and 13 knees with the diagnosis of idiopathic necrosis in the medial femoral condyle. The average age of patients was 58.3 ± 4.7 years (range 44 to 81 years). The mean BMI was 24.1±3.8 kg/m^2^ (range 22.8 to 32.4 kg/m^2^). Thirty-eight patients underwent bilateral simultaneous MIA-UKR, and 364 patients underwent unilateral MIA-UKR.

The mean length of the incisions was 5.0 ± 0.2 cm. There was 3 intraoperative complication and 4 postoperative complications. One patient had a MCL injury during surgery, but no patients had a complication of fracture during surgery. Postoperatively, 2 patients underwent arthroscopic examination 1 month post surgery due to bone cement in the joint cavity. Another 2 patients still had moderate pain in the operated knee after surgery, but no patient had a collapse of the tibial plateau, infection, thrombosis, or aseptic loosening. There were 4 patients who underwent revision surgery for dislocating their mobile bearing PE which occurred approximately 2 years after the index procedure (1.9 ± 0.3 years, range: 1.5 ~ 2.0 years); 3 of them replaced the mobile bearing PE and one patient underwent revision to total knee replacement due to radiological progression of lateral compartment OA from the initial post-operative radiographs. Thus, the survival rate was 99.1% at 73 months, and the revision rate was 0.9%.

There were significant differences between the femorotibial angle (FTA), WOMAC, VAS and KSS clinical and function scores pre and postoperatively (*p<*0.01) (Tables 1 and 2). The excellent and good rate of KSS clinical and function scores were 94.5% and 95.2% respectively.

**Table 1:** Comparing of FTA, WOMAC and VAS pre and post operation (M±SD, scores).

	FTA	WOMAC	VAS
Pre-operation	183.6°±5.1°	47.5±3.1	7.8±1.9
	(175°~189°)	(40~70)	(5~9)
Post-operation (At the last FU)	174.3°±4.2°	12.3±1.5	1.6±0.2
	(171°~184°)	(6~20)	(1~4)
*P*	0.001	0.0000	0.0000

**Table 2:** Comparing of KSS pre and post operation.

		Cases				Scores	
		Excellent	Good	Fair	Poor		
Pre-operation	Clinical Score	0	0	3	437	42.4±2.9	(35~65)
	Functional Score	0	0	22	418	53.5±3.8	(40~65)
Post-operation (the last FU)	Clinical Score	383	33	22	2	92.9±3.8	(56~98)
	Functional Score	395	24	20	1	93.5±4.0	(58~95)
*P*		#0.0000		*0. 0000		※0.0000	⋆0.0000

^#^Statistical comparing of excellent and good rate with Clinical Score pre and post operation,

*Statistical comparing of excellent and good rate with Functional Score pre and post operation,

^※^ Statistical comparing of clinical scores pre and post operation,

^⋆^Statistical comparing of functional scores pre and post operation.

Radiographic interobserver reproducibility was good, with κ = 0.65. There was no evidence of loosening in any of the 402 patients (440 knees) with follow-up radiographs. There were no pathological or complete physiological radiolucencies, but 230 patients (256 knees) had partial physiological radiolucencies (< 1 mm).

## Discussion

From the study, satisfactory midterm clinical outcomes for MIA-UKR were obtained which was comparable to the data reported by other researchers. In 2007, Aslan et.al. [11] and Kort et.al. [12] reported the results of their studies, in which patients underwent UKR with Oxford III prosthesis, and the average age of the patients was below 60 years old. The follow-up time frame ranged from 2 to 6 years. The KSS rating of good and excellent was above 90%, and the revision rate was only 0.7%. In 2013, Faour-Martín et.al. [13] reported on the clinical outcome of UKR with the Oxford III prosthesis for 402 patients (511 knees). The mean follow-up time was more than 10 years, and 96.3% of patients had an AKS score of good and excellent. In 2013, Lim HC, et.al. [14] reported the medium-term survivorship of the Oxford phase III UKR in Korean patients. They evaluated the outcome of 400 phase III Oxford UKRs in 320 Korean patients. The mean follow-up was 5.2 years. At five years, the mean KSS knee and functional scores had increased significantly. The ten-year survival rate was 94%. In 2015, Kim KT, et. al. [15] published the long-term clinical results and survival rate of minimal invasive UKR in Korea. The mean KSS knee and function scores improved significantly from 53.8 points and 56.1 points preoperatively to 85.4 points and 80.5 points at 10 year follow-up, respectively. The 10 year survival rate was 90.5% when failure was defined as all the reoperations, whereas the 10 year survival rate was 93.4% when the cases in which only revision THR was defined as failure. Yoshida K, et. al. [16] reported the Oxford UKR survival rate in Japanese patients. The study described outcomes of 1279 Oxford UKRs for Japanese patients. The mean follow-up was 5.2 years. The Oxford knee score improved from 22.3 to 40.8. The 10-year survival rate using revision was 95%. In China, Tang H, et. al. [17] evaluated the mid-term effectiveness of Oxford Phase III UKR for 26 patients (32 knees) with medial unicompartmental knee OA. The concluded the Oxford Phase III UKR had satisfactory mid-term effectiveness in treating medial unicompartmental knee OA. In this study, 402 patients (440 knees) who underwent MIA-UKR with the Oxford phrase III prosthesis completed a minimum of 60 months of follow-up. The outcome was satisfactory according to WOMAC, KSS and VAS. Patients reported good and excellent ratings in 94.5% and 95.2% of patients for their KSS clinical and function scores, respectively. The survival rate was 99.1%.

The satisfactory clinical outcomes we obtained in this study were closely related to the modern indication for UKA and proper patient selection. The modern indications of UKR were not limited to the classic indications proposed by Kozinn and Scott in 1989 [18]. Age and BMI of patients were not used to exclude patients. Mild and moderate degeneration in the patella-femoral compartment was considered acceptable.

As to patient selection for UKR, the author suggested the operation was suitable for two groups. One group was the middle and older aged population, usually no more than 60 years old. Due to the younger age and higher activity levels seen in these patients with medial compartment arthropathy, bone conserving options are preferred with TKR not being recommended as the primary treatment option. The surgery of UKR could not only solve the arthopathy in the medial compartment, restore the anatomic axis of lower limb and the arrange of patellofemoral joint, but also preserve the cruciate ligment and bone. So, after operation the patient recovered faster and the knee felt more natural. And it was also easy to be revised to TKR if needed many years later. The other group was elderly population, usually more than 80 years old. As to these patients, they usually had multiple complications and poor body conditions. As the surgery of MIA-UKR had so many advantages, such as more safety, less trauma, less bleeding, light pain, faster postoperative recovery, lower expense and shorter hospitalization time, so it was effective and much suitable for these elderly patients.

Although there are many advantages to MIA-UKR, there are disadvantages including difficult technique, smaller operative field and exposure, long learning curve, and the need for special instruments. One should keep in mind that these procedures are technically very demanding, and the long term success rate in UKR is highly correlated to the number of operations performed per year [19–27]. Weber et.al. [28] reported improved clinical outcomes with UKR if navigation was used. The authors suggested that only the surgeons who are well experienced in TKR should perform this surgery. Goodfellow [29] and Hamilton WG [20] also gave similar suggestions. In this study, there was one complication of MCL injury related to the improper handling of the saw when cutting the tibia. From this case, we learned that during surgery, care must be taken to protect the ligaments, especially the MCL and ACL.

In addition, one should always remember to not overcorrect the operated knee into too much valgus. Otherwise, overcorrection may lead to degeneration of the lateral compartment and global knee pain. In this study, 2 patients complained of moderate pain at midterm follow-up. Early in the study, much thicker mobile bearings were usually chosen for fear of dislocation, which resulted in settling the prosthesis relatively tight and potentially placing the knee into valgus. Although the overcorrection was not severe, patients still felt moderate pain in the operated knee. However, when compared to the pain prior to surgery, postoperative pain in these patients was relieved significantly, and the function significantly improvement. These patients refused to undergo revision surgery.

On the other hand, it not recommended to choose a relatively thinner PE, as this can lead to increased dislocation. In our study, there were four cases with mobile bearing dislocation who underwent surgery early in the learning curve. The three cases that underwent revision to a thicker PE had satisfactory results. For the patient who underwent revision to TKR, severe degeneration in the lateral compartment was observed, indicating that the patient may have been a candidate for TKR at the intial surgery.

As to the clinical outcome of revision after UKR, as there was only one case in this study and no long term follow-up result, so we did not have much experience for this. But according to the literatures published before, we were certain that it was very easy for UKR to be revised to TKR. And the clinical outcome of revision after UKR was comparable to the primary TKR. In 2013, O' Donnell TM, et. al. [30] reported that they compared the clinical outcome of 55 cases who received the revision operation of UKR to TKR to that of 55 cases that got the primary TKR. They concluded that there was no significant difference between the two groups at the ROM, the function of joint and radiographic evaluation.

For patients who had bone cement in the knee after UKR, a second operation was needed. We suggested that the second surgery be performed between 1–3 months after the index procedure; not earlier than 1 month for fear that the patient is too weak after UKR, and not too late for fear that the bone cement could damage the remaining cartilage and meniscus in the knee. In order to avoiding bone cement left in the joint, we suggested that one should examine the knee carefully after cementing the component and clear unnecessary bone cement thoroughly, which can be done by extending and flexing the knee joint and exposing the surgical field thoroughly. In addition, we also suggest that the tibial and femoral components be implanted separately with two separate mixing times, which may also be better for the stability of the prostheses.

No patient in this study had evidence of component subsidence or pathological radiolucency. There were 256 physiological radiolucencies and all were partial. The lines were defined as physiological radiolucent lines without clinical relevance. Tibrewal et.al. [31] distinguished between physiological radiolucent lines, which developed during the first year after surgery without any further progression, and pathological radiolucent lines caused by aseptic loosening or infection. Physiological radiolucent lines were reported by Gulati et.al. [9] in 62% of their cases. No relationship was observed between the incidence of radiolucent lines and BMI, age, gender, residual varus deformity or the status of the ACL. In this study, 58% (256/440) of cases had physiological radiolucent lines. In addition, the average FTA decreased from 183.6° preoperatively to 174.3° postoperatively according to the AP view of X-ray, and the mean rectified varus deformity angle was 9.3°. These results were satisfactory despite the high incidence of physiological radiolucent lines.

The study had several limitations. The first was that there was no comparative group included in this study, which limits the evidence of our results. However, this procedure was not very common in China when this study was being conducted, and after these promising results, we may design a prospective study comparing this technique with other well-established techniques such as osteotomy or TKR. Secondly, the study only provided midterm results. Future longer term follow-up studies should be done to further evaluate clinical outcomes of MIA-UKR. Additionally, there were 40 patients lost to follow-up in this study. This might have an affect on the result of survival rate and complication rate. Finally, this is a single-surgeon study, and the results may not be generalizable.

## Conclusion

In summary, the midterm clinical outcomes of MIA-UKR are satisfactory in a Chinese patient population, which is a good surgical option for patients with medial arthropathy of the knee. However, longer-term follow-up studies should be performed in these patients.
